# Enhanced anticancer activity of nanoemulsified cardamom extract via modulation of apoptosis- and lncRNA-associated pathways in colorectal cancer cells

**DOI:** 10.1016/j.bbrep.2026.102455

**Published:** 2026-02-01

**Authors:** Tahoora Soltani, Mohammad Karimian, Hamidreza Vaziri, Merat Karimi, Majid Nejati

**Affiliations:** a Department of Biology, Faculty of Sciences, University of Guilan, Rasht, Iran; b Department of Molecular and Cell Biology, Faculty of Basic Sciences, University of Mazandaran, Babolsar, Iran; c Institute of Nanoscience and Nanotechnology, University of Kashan, Kashan, Iran; d Anatomical Sciences Research Center, Institute for Basic Sciences, Kashan University of Medical Sciences, Kashan, Iran

**Keywords:** Cardamom nanoemulsion, Colorectal cancer, lncRNA, Gene expression, *In silico*

## Abstract

Colorectal cancer is a common, deadly disease, highlighting the need for safe and effective treatments. This study compared the anticancer effects of crude and nanoemulsified *Elettaria cardamomum* (cardamom) extract on HCT-116 colorectal cancer cells. Both the crude extract and nanoemulsion were prepared, and the nanoemulsion was characterized using transmission electron microscopy and dynamic light scattering. In an *in vitro* study, cell viability was measured by MTT assay, apoptosis by Annexin V/PI staining, and migration by scratch assay. The expression of genes related to apoptosis, migration, and angiogenesis, as well as lncRNAs MALAT1, NEAT1, and GAS5, was analyzed using real-time PCR. Physicochemical analysis showed spherical nanoemulsion particles with two size populations in a multimodal distribution. The IC_50_ was found to be 279 μg/mL for crude extract and 54.36 μg/mL for the nanoemulsion. Our molecular findings showed that the cardamom nanoemulsion exerted significantly stronger anticancer effects than the crude extract, including a greater decrease in cell viability, increased apoptosis, and more effective migration inhibition. Gene expression analysis showed that the nanoemulsion upregulated BAX and GAS5 while downregulating BCL2, MMP2, MMP9, HIF1A, VEGFA, MALAT1, and NEAT1. These expression changes were particularly pronounced at early time points compared to the crude extract. Bioinformatic analyses identified correlations between these lncRNAs and crucial genes involved in cell survival, migration, and angiogenesis, emphasizing possible lncRNA-miRNA-mRNA regulatory axes. The cardamom nanoemulsion demonstrates enhanced anticancer activity relative to the crude extract and could represent a novel therapeutic approach for colorectal cancer by modulating lncRNAs and related molecular pathways.

## Highlights

• Cardamom nanoemulsion shows stronger anticancer effects than crude extract in HCT-116 cells.
• Nanoemulsion enhances apoptosis, reduces migration, and alters key gene expression.
• lncRNAs MALAT1, NEAT1, and GAS5 modulated in response to cardamom nanoemulsion.

## 1 Introduction

Colorectal cancer is one of the most common malignancies in the world and a leading cause of cancer-related death. This disease is caused by the uncontrolled proliferation of epithelial cells in the colon or rectum, with genetic, environmental, lifestyle factors, and poor nutrition significantly contributing to its development and progression [1]. Despite significant advances in early detection and treatment, the incidence of colorectal cancer is still increasing in many regions of the world, imposing a heavy burden on healthcare systems [2]. The development and progression of colorectal cancer involve multiple, diverse, and complex molecular mechanisms [3].

In colorectal cancer, disruptions in essential cellular processes such as apoptosis and angiogenesis play a critical role in disease progression. Reduced expression of pro-apoptotic proteins like Bcl-2–associated X protein (BAX), along with increased levels of inhibitors such as B-cell lymphoma 2 (BCL2), enables cancer cells to evade programmed cell death [4]. At the same time, activation of the angiogenic pathway, marked by elevated levels of vascular-promoting factors like vascular endothelial growth factor (VEGF), regulated by hypoxia-inducible factor 1-alpha (HIF1A), which stabilizes under hypoxic conditions and enhances the transcription of pro-angiogenic genes, supports tumor growth and expansion [5,6]. Moreover, matrix metalloproteinases (MMPs) play a key role in invasion and metastasis by selectively degrading the extracellular matrix. In particular, increased levels of MMP1, MMP2, MMP3, MMP7, and MMP9 are associated with advanced stages and poor prognosis, whereas MMP12 may exert protective effects [7,8]. Additionally, long non-coding RNAs (lncRNAs) influence these pathways through epigenetic regulation and interactions with miRNAs. For instance, metastasis-associated lung adenocarcinoma transcript 1 (MALAT1) promotes migration and metastasis while inhibiting apoptosis, whereas growth arrest-specific transcript 5 (GAS5) exhibits tumor-suppressive activity by modulating the expression of BAX and BCL2 [[9], [10], [11]]. Some lncRNAs also modulate angiogenesis by affecting the HIF1A pathway. Altogether, these findings highlight the fundamental roles of these proteins and non-coding RNAs in the initiation, progression, and therapeutic responsiveness of colorectal cancer [9,12].

Colorectal cancer is treated with a combination of surgery, chemotherapy, radiotherapy, and targeted drugs, selected according to the stage and molecular profile of the tumor. Early-stage disease is mainly managed with surgery followed by adjuvant chemotherapy to reduce recurrence [13]. However, chemotherapy's systemic toxicity, harm to healthy cells, and the development of drug resistance limit its long-term effectiveness, prompting growing interest in complementary and alternative treatment approaches [1]. Herbal therapy has attracted considerable attention in recent years as a complementary treatment for colorectal cancer. Cardamom (*Elettaria cardamomum*) is one such medicinal plant, containing compounds such as cardamonin, as well as various flavonoids and terpenoids [14]. Recent studies have demonstrated that specific bioactive compounds in cardamom extract can exhibit various anticancer activities, including suppression of cell proliferation, promotion of programmed cell death, decrease of cellular migration and invasion, and inhibition of angiogenesis. These effects help restore the balance between cell growth and death and suppress tumor progression [[15], [16], [17]].

One of the main obstacles to utilizing the therapeutic properties of cardamom extract is its low bioavailability, caused by the low solubility, instability, and high volatility of its bioactive compounds in aqueous and gastrointestinal environments. These properties lead to rapid degradation or metabolism and reduced entry of active compounds into the blood and target tissues. Research has demonstrated that nanosystems like nanoemulsions can boost the bioactivity of cardamom extract by enhancing its stability, absorption, and controlled release. Therefore, nanoformulations of cardamom extract may represent an effective approach to overcome pharmacokinetic limitations and enhance its bioefficacy [[18], [19], [20]]. This study aims to compare the effects of hydroalcoholic *Elettaria cardamomum* extract and its nanoemulsion on survival, apoptosis, migration, and the expression of related genes, particularly key lncRNAs, in the human colorectal carcinoma cell line 116 (HCT-116). This research is complemented by bioinformatics approaches to provide a more comprehensive analysis of the underlying molecular processes.

## 2 Materials and methods

### 2.1 Preparation of hydroalcoholic extract

For the preparation of the hydroalcoholic extract, cardamom seeds were obtained from a local supplier. After cleaning and drying, the seeds were ground into a fine powder using an electric grinder (Chili model, Pars Khazar Co., Iran). To prepare the extraction solvent, absolute ethanol (Merck, Germany) was diluted using distilled water, and 70 % ethanol was prepared. Then, 10 g of cardamom powder was mixed with 500 mL of hydroalcoholic solvent and placed on a laboratory shaker (NOGEN Co., Iran) for 72 h at room temperature in the dark, and stirred continuously. After the maceration process was completed, the resulting mixture was filtered using Whatman No. 1 filter paper (Whatman, UK). The obtained liquid phase was subjected to a rotary evaporator (RE-5PRO, Labfreez, China) to remove the solvent and concentrate the extract. The solvent was evaporated under vacuum conditions at 39 °C with a rotation speed of 100 rpm. The resulting concentrated extract was then stored in dark glass bottles and refrigerated at 4 °C until needed.

### 2.2 Preparation and physicochemical characterization of cardamom extract nanoemulsions

To ensure full reproducibility, the nanoemulsion (NE) was formulated using a fixed ratio of components: 70 % w/w cardamom extract (concentration: 11.1 mg/mL), 10 % w/w polyethylene glycol (PEG, Merck, Germany, Cat. No. 807490) as a cosolvent, and a total of 20 % w/w surfactants composed of Tween 20 (10 % w/w; Sigma-Aldrich, Cat. No. P1379) and Tween 80 (10 % w/w; Sigma-Aldrich, Cat. No. P1754). For the preparation of 10 mL of the formulation, the corresponding volumetric equivalents were 7 mL of cardamom extract, 1 mL of PEG, 1 mL of Tween 20, and 1 mL of Tween 80. A low-energy, cost-effective method was used to prepare the NE. First, the required amount of cardamom extract was weighed into one beaker. In a separate beaker, the surfactant mixture and cosolvent were combined and then gradually added to the extract. The mixture was stirred using a magnetic stirrer (NOGEN, Co., HPN-250, Iran) at 50 °C to obtain a pre-emulsion. Subsequently, the pre-emulsion was subjected to a probe-type ultrasonicator (Hielscher UP400St Ultrasonic Processor, Germany) at 40 % amplitude for 10 min to reduce droplet size and improve stability. Sonication was performed in pulse mode (5 s on/5 s off) while the sample was immersed in an ice-water bath to maintain the temperature below 25 °C and prevent thermal degradation. The final nanoemulsion was collected in sterile containers and stored at 4 °C until use.

The physicochemical properties of the prepared nanoemulsions were evaluated using transmission electron microscopy (TEM, Zeiss EM10, Germany) to examine morphology and particle size distribution, and dynamic light scattering (DLS, VASCO2, Cordouan Tech, France) to measure particle size and polydispersity index. These techniques enabled detailed analysis of the structure and stability of the nanoemulsions. For TEM analysis, samples were prepared using a staining method with phosphotungstic acid (Sigma-Aldrich, Cat. No. P4006), suitable for studying nanoemulsions. The zeta potential, which indicates the strength of the electrostatic charge on the droplet surface, was also measured.

### 2.3 HCT-116 cell culture, monitoring, and passaging

After purchasing the HCT-116 cell line from the Pasteur Institute (Tehran, Iran), cells were first examined for density, appearance, and potential contamination using an inverted microscope (Olympus IX71, Tokyo, Japan). Cells were cultured in Dulbecco's Modified Eagle Medium (DMEM; Gibco, Cat. No. 11965084) supplemented with 10 % fetal bovine serum (FBS; Gibco, Cat. No. 26140079), 100 IU/mL penicillin, and 100 μg/mL streptomycin (Sigma-Aldrich, Cat. No. P4333), and incubated at 37 °C in a humidified atmosphere with 5 % CO_2_ and 95 % relative humidity. Flasks were inspected daily under a microscope to monitor cell growth, division, morphology, and any signs of contamination. Due to the adhesion of the cells to the bottom of the flask, the spent culture medium was first removed, then the cells were washed once with phosphate-buffered saline (PBS, Pishgam, Iran) and replaced with an appropriate amount of fresh medium. When the cells reached approximately 80 % confluency, they were passaged to continue culture and prevent overconfluency. Cell counting was performed using trypan blue staining and a Neubauer hemocytometer. For this purpose, 10 μl of the cell suspension was mixed with 10 μl of 0.4 % trypan blue (Sigma-Aldrich, Cat. No. T8154). Then, 10 μL of this mixture was carefully loaded into the chamber between the Neubauer hemocytometer (Marienfeld Superior, Cat. No. 0640010, Germany) and the coverslip, and the cells were counted under a microscope. For experimental treatments, cells were divided into four groups: the positive control group treated with doxorubicin (DOX, Sigma-Aldrich, Cat. No. D1515), Treatment 1 with hydroalcoholic cardamom extract, Treatment 2 with nanoemulsion, and the negative control group cultured in medium only.

### 2.4 Cytotoxicity assessment using MTT assay

The 3-(4,5-Dimethylthiazol-2-yl)-2,5-Diphenyltetrazolium Bromide (MTT; Sigma-Aldrich, Cat. No. M2128) assay was performed to evaluate the cytotoxicity of the hydroalcoholic extract of cardamom and its nanoemulsion on the human colorectal cancer cell line HCT-116. HCT-116 cells were seeded at a density of 10,000 cells per well in 96-well plates and incubated for 24 h to allow the cells to adhere and reach optimal conditions for treatment. The cells were subsequently treated with different doses of the extract and the nanoemulsion formulation. In addition, the cytotoxic potential of the extract-free nanoemulsion carrier (blank formulation) was evaluated using the MTT assay. Each treatment was performed in triplicate, and cells were incubated for an additional 48 h. Following treatment, the culture medium was removed, and fresh medium containing 0.5 mg/mL MTT was added to each well. Plates were incubated for 4 h at 37 °C to allow the formation of formazan crystals. The supernatant was then carefully removed, and 200 μL of dimethyl sulfoxide (DMSO; Sigma-Aldrich, Cat. No. D8418) was added to dissolve the formazan. Plates were incubated in the dark for 10 min to ensure complete solubilization. The absorbance of each well was measured at 575 nm using an enzyme-linked immunosorbent assay (ELISA) reader (EPOCH, Germany), and the percentage of cell viability was calculated relative to the untreated control wells.

To assess the selectivity of cytotoxicity, the MTT assay was also performed in a similar manner on a normal human dermal fibroblast cell line. Fibroblast cells were treated under the same culture conditions, seeding density, concentrations of the hydroalcoholic cardamom extract and its nanoemulsion, incubation times, and MTT assay procedures as described for HCT-116 cells. Cell viability percentages were calculated relative to the untreated control wells and compared with the results obtained from the HCT-116 cell line to evaluate the specificity of cytotoxic effects toward cancer cells.

### 2.5 Apoptosis assessment

Cell apoptosis was assessed 48 h after treatment with DOX, extract, and nanoemulsion, as well as in the control group, using the Annexin V-fluorescein isothiocyanate (FITC)/propidium iodide (PI) kit (BD Biosciences, cat. no. 556547) and FACSCan flow cytometry system (BD Biosciences, USA). The measurement process was performed according to the detailed protocols and instructions provided by the manufacturer to allow the separation of viable, early apoptotic, and late apoptotic cells.

### 2.6 Scratch test

Cells from the control, DOX, extract, and nanoemulsion groups were placed in controlled incubator conditions to allow their growth and proliferation under appropriate conditions. When the cell density reached about 90 %, a precise vertical scratch was created at the center of the culture wells using a 10–100 μL pipette tip to simulate a wound healing assay. At 0, 24, 48, and 72 h following the scratch, images of the wound area were captured using an inverted optical microscope equipped with a 10× objective lens to monitor cell migration. The captured images were analyzed to measure the extent of wound closure in each group. Migration was expressed as a percentage wound closure using the ImageJ tool.

### 2.7 RNA extraction, cDNA synthesis, and real-time PCR

At 24, 48, and 72 h after treatments, cells were collected, and real-time PCR was used to examine the expression of the studied genes. For this purpose, total RNA was extracted from the cells using a dedicated kit (Pars Toos, Mashhad, Iran) and converted to complementary DNA (cDNA) using another commercial kit (Pars Toos, Mashhad, Iran). All steps were performed according to the manufacturer's instructions. Then, specific primers for the studied genes, as well as the glyceraldehyde-3-phosphate dehydrogenase (GAPDH) as a reference gene, were designed using Primer3 software. For this purpose, the full-length sequences of each target gene mRNA of human origin were retrieved from the NCBI database (National Center for Biotechnology Information, https://ncbi.nlm.nih.gov). Primers were then designed, and their specificity was verified using basic local alignment search tool (BLAST). The primer sequences and their characteristics are listed in Table 1, and all primers were synthesized by Pishgam (Tehran, Iran).

**Table 1:** Sequence and characteristics of the specific primers.

Gene name	Accession no.	Forward primer (5′-3′)	GC%	Reverse prier (5′-3′)	GC%	Product size (bp)
*BAX*	NM_001291428.2	AAGAAGCTGAGCGAGTGTCT	50	GTTCTGATCAGTTCCGGCAC	55	236
*BCL2*	NM_001438935.1	CTCCTTCATCGTCCCCTCTC	60	CGGCGGCAGATGAATTACAA	50	177
*MMP2*	NM_004530.6	AATCCCACCAACCCTCAGAG	55	GTGCCCTCTTGAGACAGTCT	55	167
*MMP9*	NM_004994.3	GAGTTCCCGGAGTGAGTTGA	55	AAAGGTGAGAAGAGAGGGCC	55	225
*HIF1A*	NM_001530.4	TCCAAGAAGCCCTAACGTGT	50	TGATCGTCTGGCTGCTGTAA	50	180
*VEGFA*	NM_001171623.2	GGCCAGCACATAGGAGAGAT	55	ACGCTCCAGGACTTATACCG	55	155
*MALAT1*	NR_002819.5	CCCCTGGGCTTCTCTTAACA	55	TAGATCAAAAGGCACGGGGT	50	206
*NEAT1*	NR_028272.1	GCTACAAGGTGGGGAAGACT	55	AGTCTGACGCCCATCTTTCA	50	228
*GAS5*	NR_002578.4	GAGCAAGCCTAACTCAAGCC	55	ACACAGTGTAGTCAAGCCGA	50	207
*GAPDH*	NM_002046.7	CACATCGCTCAGACACCATG	55	TGACGGTGCCATGGAATTTG	50	198

Quantitative polymerase chain reaction (qPCR) was performed in a volume of 20 μl containing 10 μl SYBR Green (2X, Pars Toos, Mashhad, Iran), 0.5 μl each of forward and reverse primers, and 20 ng of template cDNA in a Roche LightCycler 96 real-time PCR system (Roche, Germany). Melting curve analysis was performed to ensure the accuracy of real-time PCR. The amplification protocol involved an initial denaturation at 94 °C for 5 min, followed by 35 cycles of denaturation at 94 °C for 30 s, annealing at 60 °C for 30 s, and extension at 72 °C for 30 s, ending with a final extension at 72 °C for 5 min. For gene expression analysis, the Ct value of the reference gene was subtracted from the Ct values of the target genes to obtain the ΔCt. Subsequently, the ΔCt values of the comparison groups were subtracted from each other to calculate the ΔΔCt. Finally, the resulting value was applied to equation 2^−ΔΔCt^, which represents the relative gene expression level.

### 2.8 Bioinformatics analysis

To gain a deeper understanding of the functional and regulatory interactions between genes and long non-coding RNAs in colorectal cancer, a series of bioinformatics analyses were conducted using reputable databases. In the first step, the Gene Multiple Association Network Integration Algorithm (GeneMANIA) database was used to examine the gene interaction networks between the BAX, BCL2, MMP2, MMP9, HIF1A, and VEGFA genes. This database provides information on co-expression, common signaling pathways, protein interactions, and shared functional properties among the genes of interest using machine learning algorithms and experimental datasets. Next, the Gene Expression Profiling Interactive Analysis 2 (GEPIA2) server (version 2.0; http://gepia2.cancer-pku.cn) was used to examine the relationship between the expression of lncRNAs MALAT1, NEAT1, and GAS5 with the aforementioned genes. In this analysis, the correlation coefficient between the expression of these lncRNAs and the target genes was calculated in two types of tumors: colon adenocarcinoma (COAD) and rectal adenocarcinoma (READ). GEPIA2, relying on data from the Cancer Genome Atlas (TCGA) and the Genotype-Tissue Expression (GTEx) projects, allows for accurate comparison of expression patterns in normal and tumor tissues. For the correlation analysis, GEPIA2 parameters were set as follows: exact gene symbol matching, Pearson correlation method, a *P*-value cutoff of 0.05, and combined data from the TCGA and GTEx databases. In addition, the Multiple Gene Comparison tool in GEPIA2 was used to simultaneously compare the expression levels of BAX, BCL2, MMP2, MMP9, HIF1A, and VEGFA genes in tumor and non-tumor samples to determine the differences in the expression patterns of these genes in normal and cancer tissues. For the Multiple Gene Comparison analysis, log2(TPM + 1) transformed expression data was used for plotting, with matched normal data set to TCGA tumor + TCGA normal. Finally, the Lnc2Cancer 3.0 database was used to identify interactions between the studied lncRNAs and miRNAs in COAD and READ cancers. In this analysis, interactions with a significance level below 0.05 were included to ensure that only statistically and biologically meaningful associations were considered.

### 2.9 Statistical analysis

All experiments, including MTT, wound healing, apoptosis, and real-time PCR, were performed in at least three independent biological replicates. Data are presented as the mean ± standard deviation (SD). All data from the aforementioned experiments were analyzed using one-way analysis of variance (ANOVA). Tukey's post hoc test was used for two-way comparisons between groups. A *P*-value of less than 0.05 (*P* < 0.05) was considered statistically significant. All analyses were performed using Statistical Package for the Social Sciences (SPSS) software (version 22), and the graphs were generated using Microsoft Excel.

## 3 Results

### 3.1 TEM examinations and dynamic light scattering analysis

**Fig. 1:**
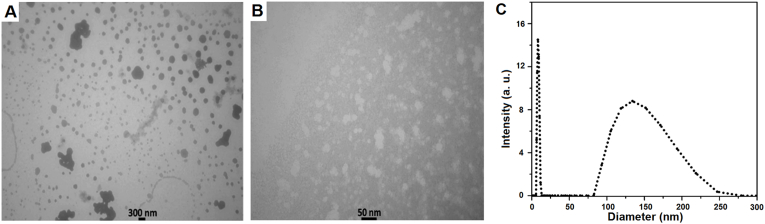
Characterization of the cardamom nanoemulsion. Transmission electron microscopy (TEM) images at scales of 300 nm (A) and 50 nm (B) reveal spherical particle morphology. Dynamic light scattering (DLS) analysis shows a multimodal particle size distribution with two predominant populations averaging ∼7.9 nm and ∼135.1 nm, respectively (C).

Observation of the nanoemulsion nanoparticles was carried out using transmission electron microscopy, which is a powerful tool for determining the structure and morphology of materials with high resolution and magnification. Fig. 1A and B shows the TEM image of the cardamom nanoemulsion sample at 50 and 300 nm scales. Nanoparticles in the figure display a spherical shape, and their measured dimensions align well with the particle sizes determined through dynamic light scattering.

The particle size of the sample was analyzed using the light scattering diffraction technique, where the results indicated that the particle sizes varied significantly among different samples. Based on the findings, the particles in the sample could be categorized into two major groups: one with an average size of approximately 7.9 nm (SD: 1.2 nm; mode: 7.7 nm; area ratio: 0.53) and the other with an average size of about 135.1 nm (SD: 31.8 nm; mode: 126.3 nm; area ratio: 0.47), reflecting a distinctly multimodal size distribution. The overall mean particle size of this sample, calculated from the results, was around 68.1 nm, which, according to the particle size distribution analysis and the calculated indices, suggests the presence of heterogeneous powdered particles with considerable dispersion. Furthermore, a polydispersity index (PDI) of 0.332, which is consistent with the broad and multimodal distribution observed and confirms a markedly polydisperse system (Fig. 1C). Also, the zeta potential was measured to be 4.4 mV, indicating weak electrostatic stability and suggesting that the physical stability of the system is mainly dependent on steric effects arising from the presence of nonionic surfactants.

### 3.2 Cytotoxicity test

**Fig. 2:**
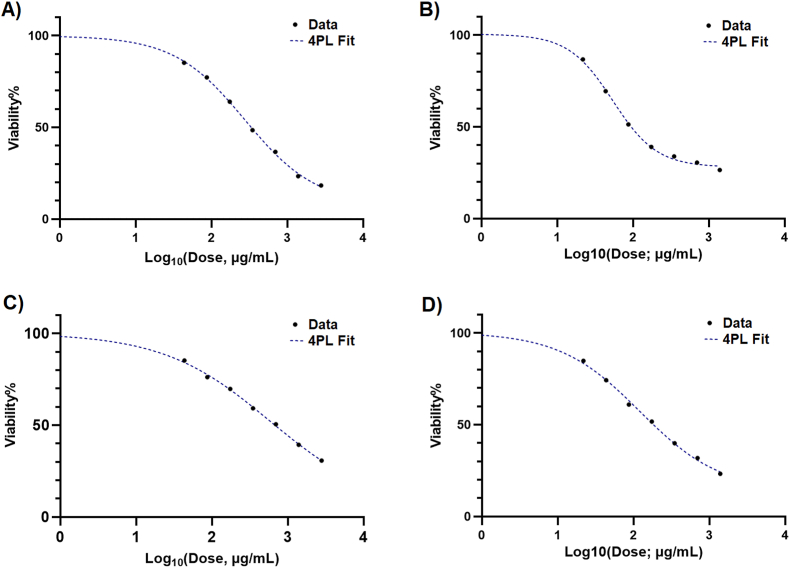
Dose-response curves obtained from MTT assays following 48 h treatment with the crude extract and its nanoemulsion formulation. Crude extract on HCT-116 cells (A), nanoemulsion on HCT-116 cells (B), crude extract on fibroblast cells, and nanoemulsion on fibroblast cells (D).

The four-parameter logistic (4 PL) model provided an excellent fit for both the crude extract and its nanoemulsion when tested on HCT-116 colorectal cancer cells following a 48-h treatment period. For the crude extract, the half-maximal inhibitory concentration (IC_50_) was calculated as 279.8 μg/mL (95 % CI: 225.7 to 371.1; Fig. 2A). The high goodness of fit (R^2^ = 0.9991) confirmed the reliability and consistency of the dose-response curve under these experimental conditions. In contrast, the nanoemulsion exhibited markedly stronger inhibitory activity in HCT-116 cells, with an IC_50_ of 54.36 μg/mL (95 % CI: 46.25 to 64.51). The goodness of fit for the nanoemulsion curve was exceptionally high (R^2^ = 0.9981; Fig. 2B).

The MTT dose-response analysis using a four-parameter variable slope model showed an IC_50_ value of 581.9 μg/mL for the extract in fibroblast cells (Fig. 2C). The model demonstrated an excellent goodness of fit (R^2^ = 0.9988), supporting the reliability of the IC_50_ estimation. The IC_50_ value of the nanoemulsion on fibroblasts was 122.1 μg/mL, with a 95 % confidence interval ranging from 85.37 to 224.4 μg/mL (Fig. 2D). The concentration-response data showed an excellent fit to the model, as indicated by a coefficient of determination of R^2^ = 0.9980. The results of the MTT assay for the blank nanoemulsion showed IC_50_ values of approximately 1.670 % v/v (95 % CI: 0.9456 to 22.26; R^2^ = 0.9980) and 1.981 % v/v (95 % CI: 1.304 to 4.935; R^2^ = 0.9980) for HCT-116 and fibroblast, respectively, indicating low cytotoxicity of the carrier components at the tested concentrations.

Overall, the nanoemulsion demonstrated substantially enhanced cytotoxic activity against HCT-116 colorectal cancer cells compared with the crude extract. Importantly, despite this increased anticancer efficacy, the nanoemulsion showed considerably higher IC_50_ values in fibroblast cells, indicating a relative selectivity toward cancer cells (selectivity index> 2). Moreover, the minimal cytotoxicity observed for the blank nanoemulsion confirms that the biological effects are primarily attributable to the active formulation rather than the carrier system.

### 3.3 Apoptosis analysis by Annexin V-FITC/PI staining

**Fig. 3:**
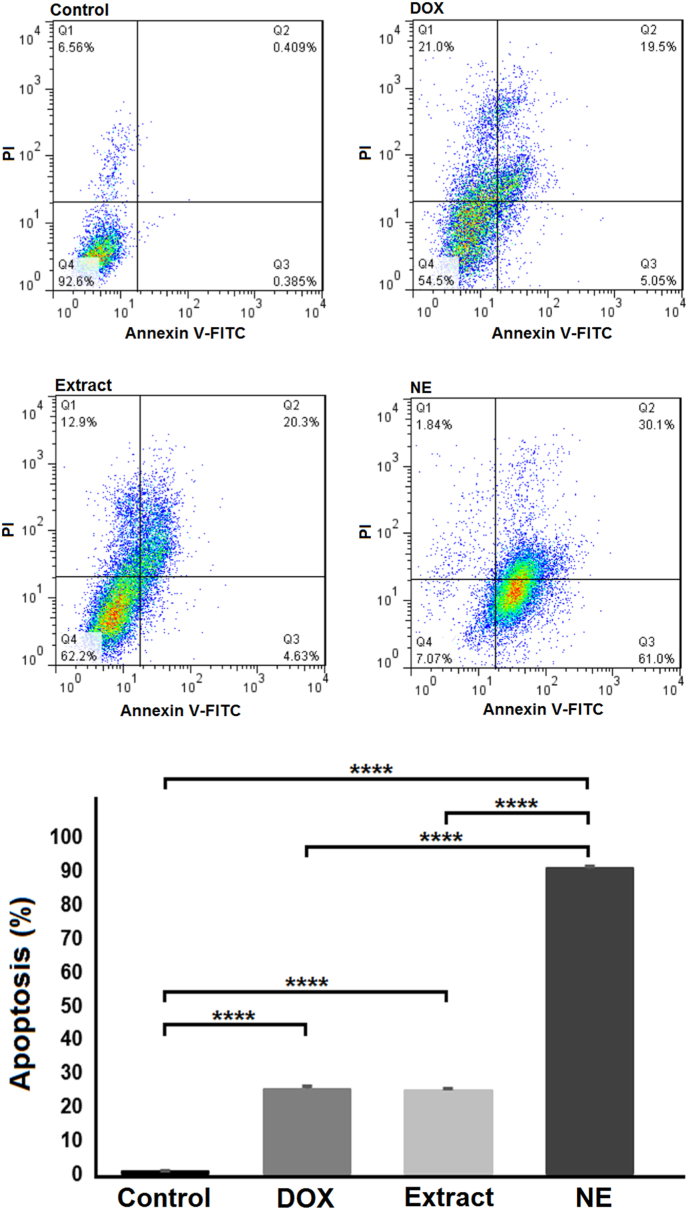
Flow cytometric analysis using the Annexin V-FITC assay to evaluate apoptosis in cells treated with DOX, cardamom extract, and nanoemulsion, compared to the control group. Apoptosis was significantly elevated in all treated groups compared to the control. Even the nanoemulsion group exhibited a notably higher apoptotic rate than both the DOX and extract groups. Data are presented as mean ± standard deviation (n = 3). ∗∗∗∗ denotes *P* < 0.0001. DOX: doxorubicin; NE: nanoemulsion.

Annexin V-FITC/PI assay was used to determine programmed cell death. In this method, after culturing and treating cells with cardamom extract, nanoemulsion, as well as in the untreated negative groups and the DOX positive control group, the rate of early and delayed apoptosis was measured (Fig. 3). Flow cytometry analysis revealed a significant increase in both early and late apoptosis rates in the DOX, extract, and nanoemulsion-treated groups compared to the untreated control group (*P* < 0.0001). As shown in Fig. 3, in the DOX and extract groups, the rate of apoptosis increased by about 25 %. However, in the nanoemulsion-treated group, apoptosis induction was markedly higher, reaching nearly 90 %, which was significantly higher than both the extract and DOX groups (*P* < 0.0001).

### 3.4 Cell migration analysis in the extract and nanoemulsion-treated groups

**Fig. 4:**
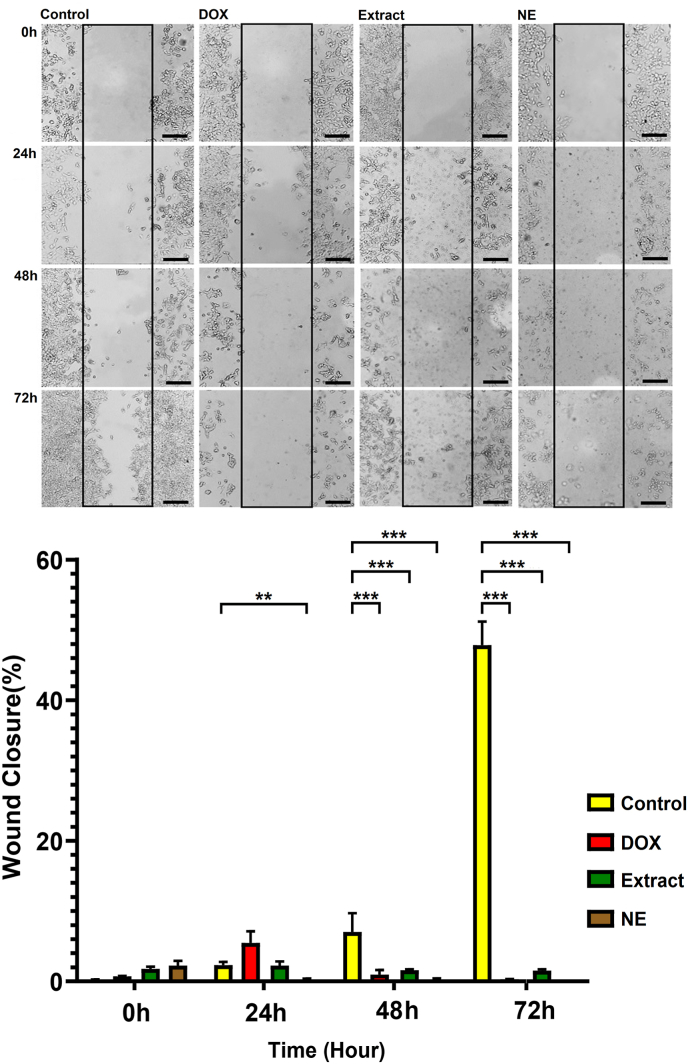
Scratch test results. No significant differences were observed at 0 h; however, wound closure was significantly reduced in the nanoemulsion group at 24 h and in all treated groups at 48 and 72 h compared with the control group. Data are shown as mean ± standard deviation (n = 3). ∗∗ and ∗∗∗ indicate *P* < 0.01 and *P* < 0.001, respectively. Black scale bars indicate a length of 200 μm; Magnification: 100X. DOX: doxorubicin; NE: nanoemulsion.

Following treatment of the cells with DOX, cardamom extract, and nanoemulsion, cell migration was assessed at 24, 48, and 72 h using the scratch assay and compared to the control group. Fig. 4 illustrates that at time zero, no significant difference was detected between the groups. After 24 h, no significant difference was observed between the DOX and extract groups compared to the control group; however, the percentage of wound closure in the nanoemulsion group showed a significant decrease compared to the control group (*P* < 0.01). At 48 h, the percentage of wound closure in the DOX, extract, and nanoemulsion groups showed a significant decrease compared with the control group (*P* < 0.001). Similarly, at 72 h, all three groups (DOX, extract, and nanoemulsion) exhibited a significant reduction in wound closure percentage compared with the control group (*P* < 0.001).

### 3.5 Changes in the expression of genes involved in apoptosis, migration, and angiogenesis

**Fig. 5:**
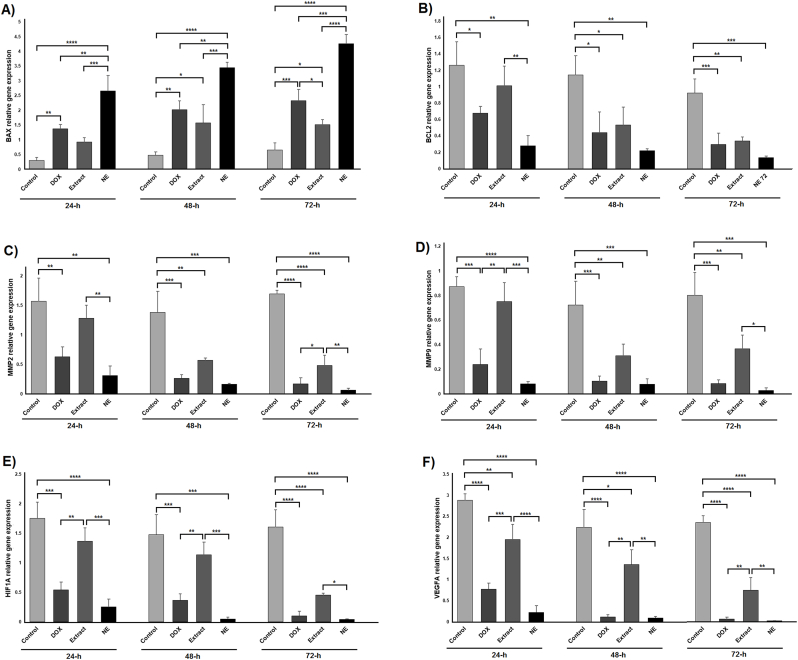
Data from the expression of the studied genes. Changes in gene expression of BAX (A), BCL2 (B), MMP2 (C), MMP9 (D), HIF1A (E) and VEGFA (F) in different groups and at 24, 48 and 72 h after treatments. Data are shown as mean ± standard deviation (n = 3). ∗, ∗∗, ∗∗∗ and ∗∗∗∗ indicate *P* < 0.05, *P* < 0.01, *P* < 0.001, and *P* < 0.0001, respectively. DOX: doxorubicin; NE: nanoemulsion.

After completing the treatments, the expression levels of apoptosis-related genes (BAX and BCL2), migration-associated genes (MMP2 and MMP9), and angiogenesis-related genes (HIF1A and VEGFA) were assessed in the various groups at three time intervals: 24, 48, and 72 h. Our results demonstrated that BAX expression in the nanoemulsion group was significantly elevated compared to the control group at all three time points (24, 48, and 72 h; *P* < 0.0001). Conversely, BAX expression in the cardamom extract group showed a significant rise only at 48 and 72 h relative to the control group (*P* < 0.05). A comparison between the extract and nanoemulsion groups with the positive control (DOX) group indicated that BAX expression in the nanoemulsion group was considerably higher at 24 h (*P* < 0.01), 48 h (*P* < 0.01), and 72 h (*P* < 0.001). However, BAX expression in the extract group was significantly lower compared to the DOX group at 72 h (*P* < 0.05). Additionally, BAX expression in the nanoemulsion group was markedly greater than in the extract group at all three time points: 24 h (*P* < 0.001), 48 h (*P* < 0.001), and 72 h (*P* < 0.0001) (Fig. 5A). The findings for BCL2 gene expression alterations are illustrated in Fig. 5B. The data indicated that BCL2 expression in the nanoemulsion group was significantly decreased compared to the control group at 24 h (*P* < 0.01), 48 h (*P* < 0.01), and 72 h (*P* < 0.001). In the extract group, BCL2 expression exhibited a significant decline only at 48 h (*P* < 0.05) and 72 h (*P* < 0.01) relative to the control. Moreover, at 24 h, BCL2 levels in the nanoemulsion group were significantly lower than those observed in the cardamom extract group (*P* < 0.01).

The expression profiles of MMP2 and MMP9, two critical genes associated with cell migration, are presented in Fig. 5C and D, respectively. Our findings showed that MMP2 expression in the nanoemulsion group was significantly decreased compared to the control group at 24 h (*P* < 0.01), 48 h (*P* < 0.001), and 72 h (*P* < 0.0001). Conversely, in the cardamom extract group, MMP2 expression exhibited a significant decline only at 48 h (*P* < 0.01) and 72 h (*P* < 0.0001) relative to the control. A comparison between the nanoemulsion and extract groups revealed that MMP2 expression was significantly lower in the nanoemulsion group at both 24 h and 72 h (*P* < 0.01). Furthermore, MMP2 expression in the extract group was significantly elevated compared to the DOX group at 72 h (*P* < 0.05). Concerning MMP9, the results demonstrated a significant downregulation of its expression in the nanoemulsion group compared to the control at 24 h (*P* < 0.0001), 48 h (*P* < 0.001), and 72 h (*P* < 0.001). Additionally, MMP9 expression in the extract group was significantly reduced compared to the control at 48 h and 72 h (*P* < 0.01). Comparison between the two treatments showed that MMP9 expression in the nanoemulsion group was significantly reduced compared to the extract group at 24 h (*P* < 0.001) and 72 h (*P* < 0.05). Notably, MMP9 expression in the extract group was significantly higher than in the positive control (DOX) at 24 h (*P* < 0.01).

The expression levels of HIF1A and VEGFA, two critical genes involved in angiogenesis, were also assessed across the different groups, with results shown in Fig. 5E and F, respectively. Our data revealed that HIF1A expression in the nanoemulsion group was significantly suppressed compared to the control group at 24 h (*P* < 0.0001), 48 h (*P* < 0.001), and 72 h (*P* < 0.0001). In the cardamom extract group, however, a significant decrease in HIF1A expression was detected only at 72 h relative to the control (*P* < 0.0001). Moreover, HIF1A levels in the nanoemulsion group were significantly lower than those in the extract group at 24 h (*P* < 0.001), 48 h (*P* < 0.001), and 72 h (*P* < 0.05). Conversely, HIF1A expression in the extract group was significantly elevated compared to the DOX positive control at 24 h and 48 h (*P* < 0.01). Analysis of VEGFA expression demonstrated a marked downregulation in the nanoemulsion group relative to the control at all treatment time points (*P* < 0.0001). Similarly, VEGFA levels in the extract group were significantly reduced compared to controls at 24 h (*P* < 0.01), 48 h (*P* < 0.05), and 72 h (*P* < 0.0001). Furthermore, VEGFA expression in the nanoemulsion group was significantly lower than in the extract group at 24 h (*P* < 0.0001), 48 h (*P* < 0.01), and 72 h (*P* < 0.01). Interestingly, VEGFA expression in the extract group was significantly higher than in the DOX group at 24 h (*P* < 0.001), 48 h (*P* < 0.01), and 72 h (*P* < 0.01).

### 3.6 Expression changes of long noncoding RNAs

**Fig. 6:**
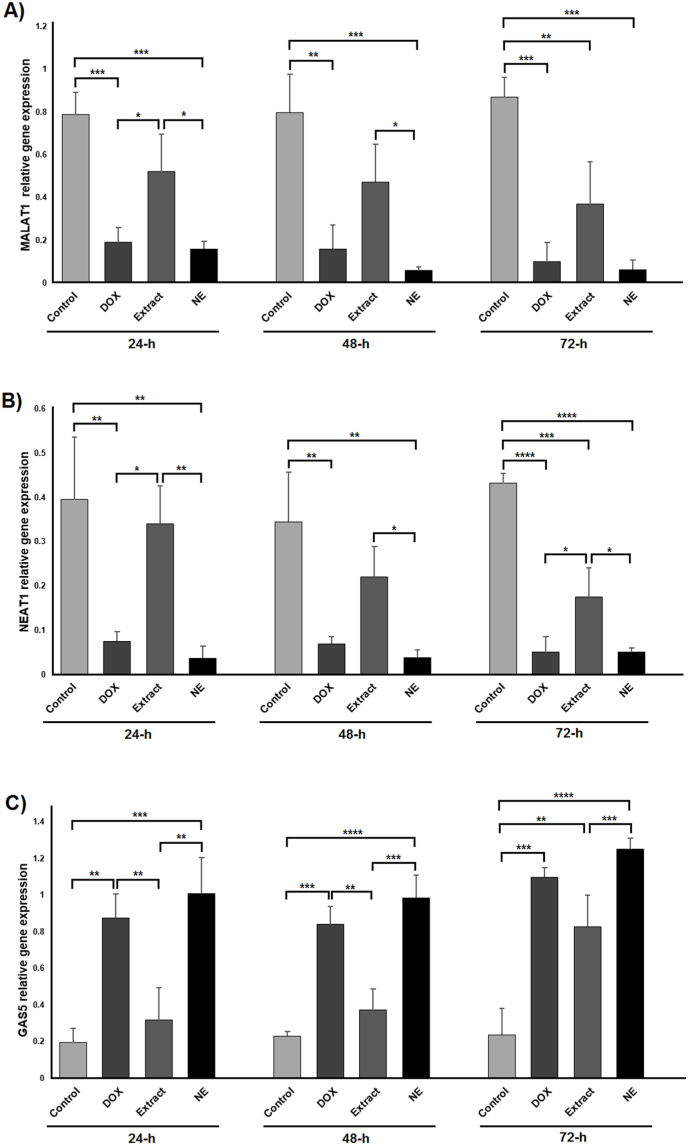
Data from the expression of the studied lncRNAs. Changes in the expression of long non-coding RNAs MALAT1 (A), NEAT1 (B), and GAS5 (C) in different groups and at 24, 48, and 72 h after treatments. Data are shown as mean ± standard deviation (n = 3). ∗, ∗∗, ∗∗∗ and ∗∗∗∗ indicate *P* < 0.05, *P* < 0.01, *P* < 0.001 and *P* < 0.0001, respectively. DOX: doxorubicin; NE: nanoemulsion.

The expression levels of the long noncoding RNAs MALAT1, NEAT1, and GAS5 were assessed using real-time PCR, with results summarized in Fig. 6. MALAT1 expression was significantly reduced in the nanoemulsion group compared to the control group at 24 h, 48 h, and 72 h (*P* < 0.001). Conversely, MALAT1 expression in the extract group showed a significant decline only at 72 h relative to the control (*P* < 0.01). Additionally, comparison between the extract and nanoemulsion groups indicated that MALAT1 was significantly downregulated in the nanoemulsion group at both 24 h and 48 h (*P* < 0.05). Notably, MALAT1 expression in the extract group was significantly increased compared to the DOX group at 24 h (*P* < 0.05; Fig. 6A).

The expression patterns of NEAT1 across different groups and time points are illustrated in Fig. 6B. As depicted, NEAT1 expression in the nanoemulsion group was significantly decreased compared to the control group at 24 h (*P* < 0.01), 48 h (*P* < 0.01), and 72 h (*P* < 0.0001). In contrast, the extract group showed a significant reduction in NEAT1 expression only at 72 h relative to the control (*P* < 0.001). Furthermore, NEAT1 levels in the nanoemulsion group were significantly lower than those in the extract group at 24 h (*P* < 0.01), 48 h (*P* < 0.05), and 72 h (*P* < 0.05). Additionally, NEAT1 expression in the extract group was significantly elevated compared to the DOX group at both 24 h and 72 h (*P* < 0.05).

Details of GAS5 expression are presented in Fig. 6C. GAS5 was significantly upregulated in the nanoemulsion group compared to the control group at 24 h (*P* < 0.001), 48 h (*P* < 0.0001), and 72 h (*P* < 0.0001). In contrast, the extract group exhibited a significant increase in GAS5 expression only at 72 h relative to the control (*P* < 0.01). Moreover, GAS5 levels were significantly higher in the nanoemulsion group than in the extract group at all examined time points: 24 h (*P* < 0.01), 48 h (*P* < 0.001), and 72 h (*P* < 0.001). Additionally, GAS5 expression in the extract group was significantly reduced compared to the DOX group at 24 h and 48 h (*P* < 0.01).

### 3.7 Bioinformatics results

**Fig. S1:**
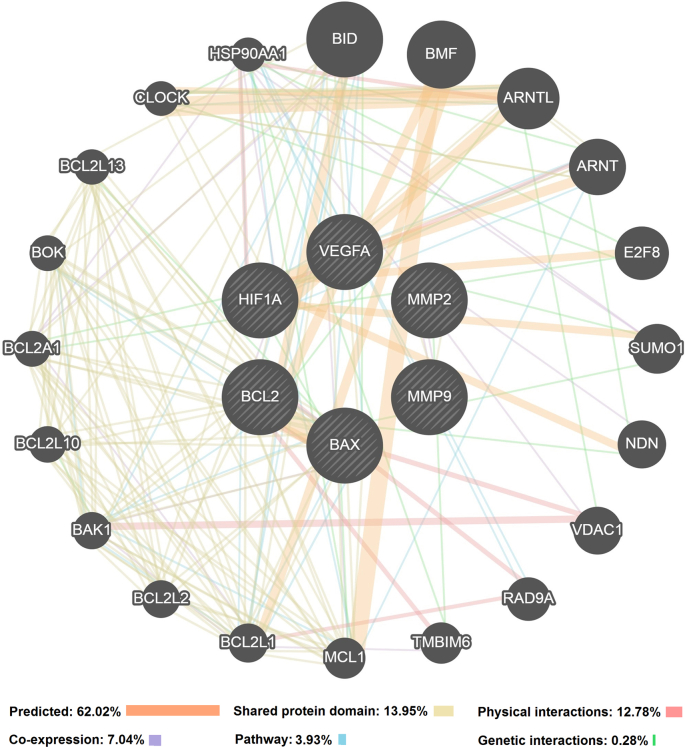
Results obtained from GeneMANIA. As shown, the six genes BAX, BCL2, MMP2, MMP9, HIF1A, and VEGFA interact with a set of 20 other genes. Analysis of the interaction types revealed that the majority of them (62.02 %) were predicted interactions, which are indicated by orange lines.

Network analysis using GeneMANIA (Fig. S1) revealed that the six studied proteins BAX, BCL2, MMP2, MMP9, HIF1A, and VEGFA interact with 20 other proteins. Among the various types of interactions, predicted interactions (highlighted in orange) represented the majority, comprising 62.02 % of the total. Shared protein domains represented 13.95 % of interactions, while physical interactions (pink) and co-expression (purple) constituted 12.78 % and 7.04 % of total interactions, respectively. In this network, BAX, BCL2, and HIF1A exhibited the greatest connectivity, while MMP2, MMP9, and VEGFA demonstrated comparatively fewer interactions.

**Fig. 7:**
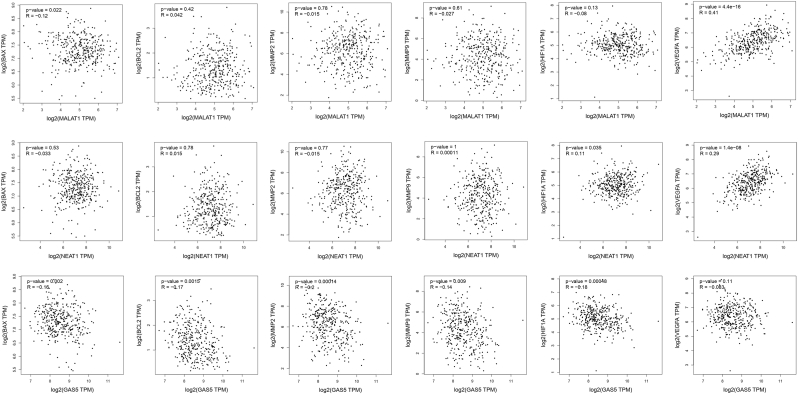
Correlation of MALAT1, NEAT1, and GAS5 expression with the expression of BAX, BCL2, MMP2, MMP9, HIF1A, and VEGFA. The correlations of MALAT1 with BAX and VEGFA (first row), NEAT1 with HIF1A and VEGFA (second row), and GAS5 with BAX, BCL2, MMP2, MMP9, and HIF1A (third row) are shown.

**Fig. S2:**
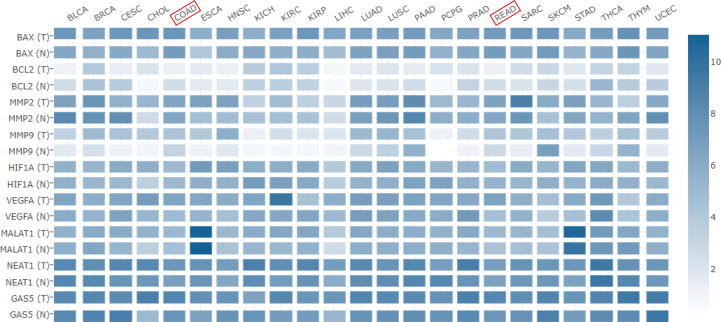
Expression of BAX, BCL2, MMP2, MMP9, HIF1A, VEGFA, MALAT1, NEAT1, and VEGFA genes in tumor and normal tissues. As it is clear, the studied genes are more expressed in COAD and READ tumor tissues than in normal samples. The column of COAD and READ tumor tissues is shown with a red box.

Next, we analyzed how the expression of the lncRNAs MALAT1, NEAT1, and GAS5 correlates with the above-mentioned genes in the two tumor types, COAD and READ. The results indicated that elevated MALAT1 expression was significantly associated with increased VEGFA expression (*P* = 4.4e-16, R = 0.41), while showing a significant negative correlation with BAX expression (*P* = 0.022, R = −0.12). NEAT1 expression was positively correlated with both HIF1A (*P* = 0.035, R = 0.11) and VEGFA (*P* = 1.4e-08, R = 0.29). For GAS5, elevated expression was significantly linked to lower expression of BAX (*P* = 0.002, R = −0.16), BCL2 (*P* = 0.0015, R = −0.17), MMP2 (*P* = 0.00014, R = −0.20), MMP9 (*P* = 0.009, R = −0.14), and HIF1A (*P* = 0.00048, R = −0.18) (Fig. 7). Using the Multiple Gene Comparison tool in GEPIA2, the expression of BAX, BCL2, MMP2, MMP9, HIF1A, and VEGFA was compared between tumor and normal samples. The results demonstrated that most of these genes were expressed at higher levels in COAD and READ tumor tissues compared to normal tissues (Fig. S2). It should be noted that despite statistical significance in several associations, some correlations (e.g., |R| < 0.2) represent weak effect sizes. These weak correlations likely reflect subtle regulatory tendencies rather than strong biological interactions and should therefore be interpreted with caution.

**Fig. S3:**
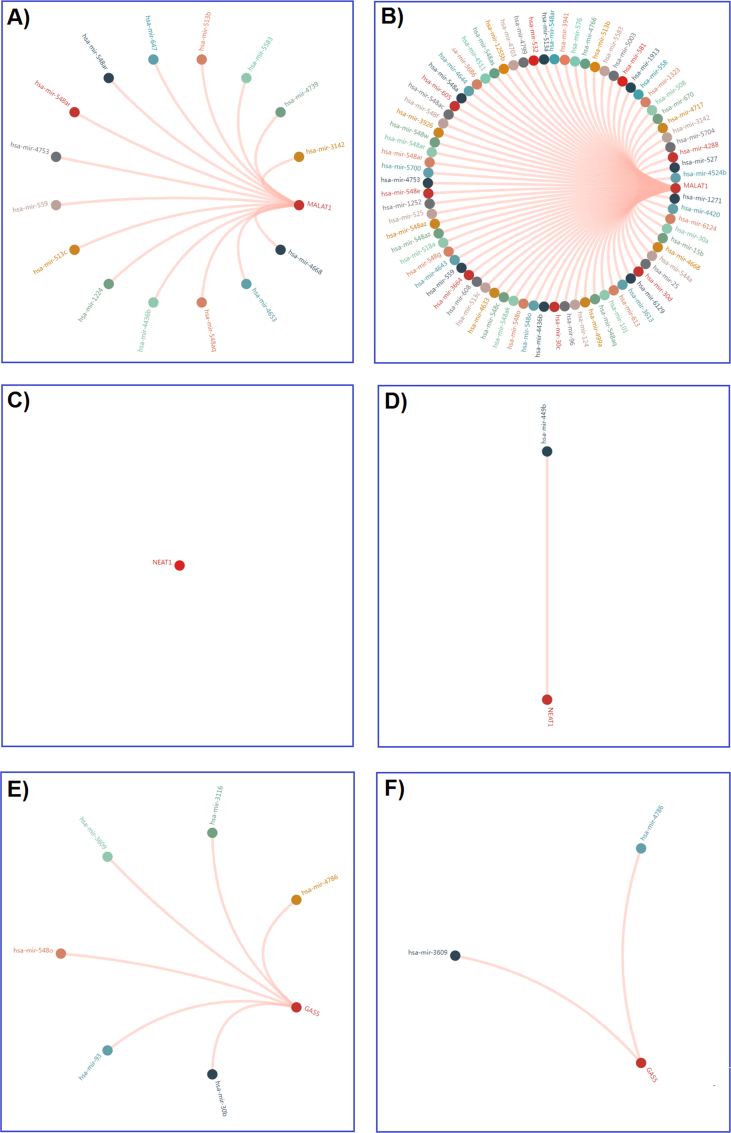
Interaction of MALAT1, NEAT1, and GAS5 with miRNAs in READ and COAD tumors. Data from lnc2Cancer 3.0 showed that MALAT1 interacts with 15 miRNAs in READ tumors (A) while interacting with a larger number of miRNAs in COAD tumors (B). However, data from NEAT1 showed that this lncRNA interacts with no miRNAs in READ tumors (C) and only one miRNA in COAD tumors (D). Data from GAS5 also showed that this lncRNA interacts with 6 miRNAs in READ tumors (E) and only 2 miRNAs in COAD tumors (F).

Finally, data obtained from Lnc2Cancer 3.0 were used to identify the interactions of the studied lncRNAs with miRNAs in COAD and READ cancers. MALAT1 was shown to interact with 15 miRNAs in READ tumors (*P* < 0.05), and with an even greater number of miRNAs in COAD tumors. NEAT1, however, showed interaction with only one miRNA, exclusively in COAD tumors. GAS5 interacted with 6 miRNAs in READ and with only 2 miRNAs in COAD (Fig. S3). Further analysis revealed that most of these miRNAs are involved in various processes related to cancer progression.

## 4 Discussion

This study compares the effects of hydroalcoholic *Elettaria cardamomum* extract and its nanoemulsion on viability, apoptosis, migration, and key lncRNA expression in HCT-116 colorectal cancer cells. The nanoemulsion showed stronger cytotoxicity, greater apoptosis induction, and more effective migration inhibition than the crude extract. The nanoemulsion had a significantly lower IC_50_, reflecting enhanced anticancer activity due to nanoformulation. Surfactants such as Tween 20, Tween 80, and PEG have been shown to influence cell viability, with some reports indicating cytotoxic effects and others demonstrating safety at low concentrations [21,22]. To ensure that the vehicle did not interfere with our observations, we evaluated the blank extract carrier, containing PEG and the surfactants, on fibroblast and HCT-116 cells. The carrier was well-tolerated at the applied doses, allowing confident interpretation of the effects attributed to the extract-loaded nanoemulsion.

Apoptosis analysis in the present study demonstrated that cardamom extract, particularly in its nanoemulsion form, significantly enhanced programmed cell death. Flow cytometry analysis revealed a significant elevation in apoptosis in cells treated with both the extract and its nanoemulsion. These findings were further corroborated by gene expression analysis, which revealed a time-dependent upregulation of BAX and downregulation of BCL2 in the treated groups. The mechanisms underlying cardamom-induced apoptosis remain unclear, but evidence indicates that cardamom can induce cell-cycle arrest and apoptosis through reactive oxygen species (ROS)-dependent pathways and c-Jun N-terminal kinase (JNK) signaling activation [23]. A likely mechanism is ROS-mediated JNK activation, which increases BAX, decreases BCL2, and triggers apoptosis [[24], [25], [26]]. Overall, the ROS–JNK cascade appears to be a key pathway in cardamom-induced apoptosis, although further studies are needed to fully clarify this process.

Cell migration assays showed that both cardamom extract and its nanoemulsion significantly inhibited cancer cell migration, as confirmed by wound healing assays and the reduced expression of MMP2 and MMP9. These metalloproteinases are major regulators of cancer cell migration and invasion [27], and their elevated activity has been reported in colorectal cancer tissues [28,29]. Earlier studies found that cardamom essential oil suppresses migration by inhibiting MMP9 [30]. Eucalyptol, a cardamom-derived monoterpenoid, can reduce MMP2 and MMP9 activity and modulate the phosphoinositide 3-kinase (PI3K)/protein kinase B (Akt)/mammalian target of rapamycin (mTOR) pathway, thereby inhibiting cancer cell migration and invasion [31]. Dysregulation of this pathway is associated with cancer progression, invasion, and metastasis [32,33], and its activation can upregulate MMP2 and MMP9 in cancer cells [32,34,35]. Altogether, these findings indicate that cardamom and its bioactive compounds may function as anti-metastatic agents by suppressing MMP expression through inhibition of the PI3K/Akt/mTOR signaling pathway.

The results of this study regarding the expression of HIF1A and VEGFA, which are key regulators of angiogenesis, demonstrated that both cardamom extract and its nanoemulsion were capable of downregulating these genes. This suppressive effect was stronger in the nanoemulsion group and increased progressively over time. A recent study reported a novel function for cardamonin, showing that it exerts its anticancer effects by inhibiting the HIF1A pathway and reprogramming HIF1A-dependent metabolic processes. The mTOR/70 kDa ribosomal protein S6 kinase (p70S6K) pathway has also been identified as a key target of cardamonin in suppressing the HIF1A/pyruvate dehydrogenase kinase 1 (PDHK1) axis [36]. Cardamonin significantly inhibited VEGF-induced angiogenesis, particularly through the downregulation of miR-21 in human endothelial cells, and restoration of miR-21 reversed the antiproliferative and anti-migratory effects of cardamonin. This suggests that modulation of miR-21 is a critical mechanism by which cardamonin suppresses tumor angiogenesis [37]. In a hepatocellular carcinoma model, plant-derived compounds such as 1,8-cineole and ellagic acid were able to reduce VEGF and MMP9, along with other markers of tumor aggressiveness. These changes were accompanied by tissue-level improvements, suggesting that these compounds can help limit cancer progression by downregulating key angiogenic factors [38]. Overall, these findings suggest that cardamom and its bioactive components can suppress tumor angiogenesis through multiple mechanisms, including modulation of HIF1A and inhibition of VEGF signaling. Consequently, cardamom emerges as a promising natural agent with considerable potential for cancer prevention and therapy via anti-angiogenic mechanisms.

In HCT-116 cells treated with cardamom, MALAT1 and NEAT1 expression decreased, while GAS5 expression increased. NEAT1 acts as an endogenous competing lncRNA that sponges tumor-suppressive miRNAs to promote tumorigenesis [39]. In hepatocellular carcinoma, elevated NEAT1 expression is accompanied by reduced miR-125a-5p levels, and NEAT1 knockdown inhibits proliferation, induces apoptosis, and suppresses angiogenesis through downregulation of the AKT/mTOR and extracellular signal-regulated kinase (ERK) pathways. Additionally, NEAT1 acts as a ceRNA for miR-125a-5p, enhancing VEGF expression and promoting angiogenesis [40]. Another study suggested that MALAT1 promotes angiogenesis via regulation of the miR-150-5p/VEGFA signaling axis [41]. MALAT1 promotes cell proliferation, inhibits apoptosis, and supports tumor growth in various cancer cell lines, highlighting its potential as a therapeutic target [42]. Conversely, GAS5 has been shown to suppress gastric cancer cell proliferation independently of p53 through the ataxia telangiectasia mutated (ATM)/p38 mitogen-activated protein kinase (MAPK) signaling pathway [43]. Cells with suppressed GAS5 fail to undergo G1/S cell cycle arrest [44]. Overall, the downregulation of NEAT1 and MALAT1, along with upregulation of GAS5 in cardamom-treated cells, likely drives reduced proliferation, apoptosis induction, and angiogenesis inhibition, highlighting cardamom's potential to modulate cancer-related pathways and offer new therapeutic opportunities.

Nanoemulsions, due to their nanoscale dimensions, are able to enter cells via endocytotic pathways and gradually release their drug payload within endosomes and lysosomes [45,46]. The very small size of nanoemulsions, significantly increases the interfacial area between phases, which in turn enhances drug dissolution, improves solubility, and increases the bioavailability of poorly water-soluble drugs, including herbal bioactives [47,48]. In classical emulsification processes, surfactants play a dual role; on the one hand, by reducing interfacial tension, they facilitate droplet deformation and breakup, and on the other hand, by preventing droplet aggregation and coalescence, they contribute to system stability. Overall, surfactants play a key role in stabilizing nanoemulsions by maintaining their physical stability and preventing the degradation of encapsulated bioactive compounds during processing and storage. Enhanced stability of nanoemulsions also leads to prolonged circulation time in the bloodstream and ultimately improves the systemic bioavailability of the compounds [49,50]. In addition, nanoemulsions can exploit the enhanced permeability and retention (EPR) effect to achieve targeted drug delivery [51]. The EPR effect refers to the preferential accumulation of nanocarriers in inflamed or tumor tissues, which arises from the high permeability of blood vessels and deficiencies in the lymphatic drainage system in these regions. This form of passive targeting increases drug concentration at the target site while simultaneously reducing unintended distribution to healthy tissues [52]. The combined effect of these synergistic processes, including improved solubility, enhanced stability, increased bioavailability, improved cellular uptake, and selective accumulation in tumor tissue, constitutes a coherent mechanism that ultimately leads to a marked enhancement of the anticancer activity of nanoemulsions compared with conventional forms of the compounds.

This study has some limitations that should be acknowledged. One of the limitations of this study is that not all complementary assays were performed for the extract-free nanoemulsion. Specifically, evaluations such as apoptosis assays, cell migration, and gene expression analyses were conducted only for the extract-loaded samples. The absence of these tests for the blank nanoemulsion limits a more precise comparison of the intrinsic effects of the nanoemulsion system with those of the extract-containing formulations. Performing these experiments on the blank sample in future studies is recommended to complete the biological evaluation. Also, it lacks *in vivo* evidence and functional assays related to the examined lncRNAs. The observed correlations between lncRNAs and their target genes reflect association only. In the absence of functional studies, such as knockdown or overexpression, any proposed mechanisms involving lncRNA-miRNA-mRNA interactions remain speculative and should be interpreted cautiously.

## 5 Conclusion

The current study demonstrated that the cardamom nanoemulsion exhibits significantly stronger anticancer impacts on HCT-116 cells compared to the crude extract. These effects included decreased cell viability, increased apoptosis induction, and more efficient inhibition of cell migration. Observed alterations in gene expression suggested that the nanoemulsion influences multiple molecular pathways by upregulating pro-apoptotic genes while downregulating genes and lncRNAs involved in cell survival, angiogenesis, and migration. These alterations were especially evident at early time points, indicating a faster and more potent effect of the nanoemulsion relative to the crude extract. Additionally, bioinformatic analyses emphasized the potential role of lncRNA-miRNA-mRNA networks in modulating pathways related to colorectal cancer development and progression. Overall, these findings suggest that nanoformulated cardamom extract represents a novel and promising approach to improve the efficacy of plant-derived compounds and may serve as an adjunctive strategy in cancer treatment.

## CRediT authorship contribution statement

Mohammad Karimian: Conceptualization, Supervision, Data analysis, Writing – original draft. Hamidreza Vaziri: Conceptualization, Supervision. Majid Nejati: Investigation, Data analysis. Tahoora Soltani: Investigation, Writing – original draft. Merat Karimi: Investigation. All authors: Writing – review & editing, final approval of the manuscript.

## Ethical approval

N/A.

## Funding information

This study was conducted with personal funding.

## Declaration of competing interest

The authors declare that they have no known competing financial interests or personal relationships that could have appeared to influence the work reported in this paper.

## Data Availability

The data generated and reported in this study are available from the corresponding author upon reasonable request.

## Abbreviations

4 PL: Four-parameter logistic, ANOVA: Analysis of variance, ATM: Ataxia telangiectasia mutated, BAX: Bcl-2–associated X protein, BCL2: B-cell lymphoma 2, cDNA: Complementary DNA, COAD: Colon adenocarcinoma, DLS: dynamic light scattering, DMEM: Dulbecco's Modified Eagle Medium, DMSO: Dimethyl sulfoxide, DOX: Doxorubicin, ELISA: Enzyme-linked immunosorbent assay, ERK: Extracellular signal-regulated kinase, FBS: Fetal bovine serum, FITC: Fluorescein isothiocyanate, GAPDH: Glyceraldehyde-3-phosphate dehydrogenase, GAS5: Growth arrest-specific transcript 5, GeneMANIA: Gene Multiple Association Network Integration Algorithm, GEPIA2: Gene Expression Profiling Interactive Analysis 2, GTEx: Genotype-Tissue Expression, HCT-116: Human colorectal carcinoma cell line 116, HIF1A: Hypoxia-inducible factor 1-alpha, JNK: Jun N-terminal kinase, lncRNAs: Long non-coding RNAs, MALAT1: Metastasis-associated lung adenocarcinoma transcript 1, MAPK: Mitogen-activated protein kinase, MMP: Matrix metalloproteinase, mTOR: Mammalian target of rapamycin, MTT: 3-(4,5-Dimethylthiazol-2-yl)-2,5-Diphenyltetrazolium Bromide, NCBI: National Center for Biotechnology Information, p70S6K: 70 kDa ribosomal protein S6 kinase, PBS: phosphate-buffered saline, PDHK1: Pyruvate dehydrogenase kinase 1, PDI: Polydispersity index, PI: Propidium iodide, PI3K: Phosphoinositide 3-kinase, qPCR: Quantitative polymerase chain reaction, READ: Rectal adenocarcinoma, ROS: Reactive oxygen species, SD: Standard deviation, SPSS: Statistical Package for the Social Sciences, TCGA: The Cancer Genome Atlas, TEM: Transmission electron microscopy, VEGF: Vascular endothelial growth factor.
