# Supplementary material for: Enhanced anticancer activity of nanoemulsified cardamom extract via modulation of apoptosis- and lncRNA-associated pathways in colorectal cancer cells
Source: Biochem Biophys Rep. 2026 Feb 1;45:102455. doi: 10.1016/j.bbrep.2026.102455 (PMC12882662; doi:10.1016/j.bbrep.2026.102455)
Supplement: Multimedia component 1 [file mmc1.pdf]

Q1: Necrosis

Q2: Late Apoptosis

Q3: Early Apoptosis

Q4: Live Cells

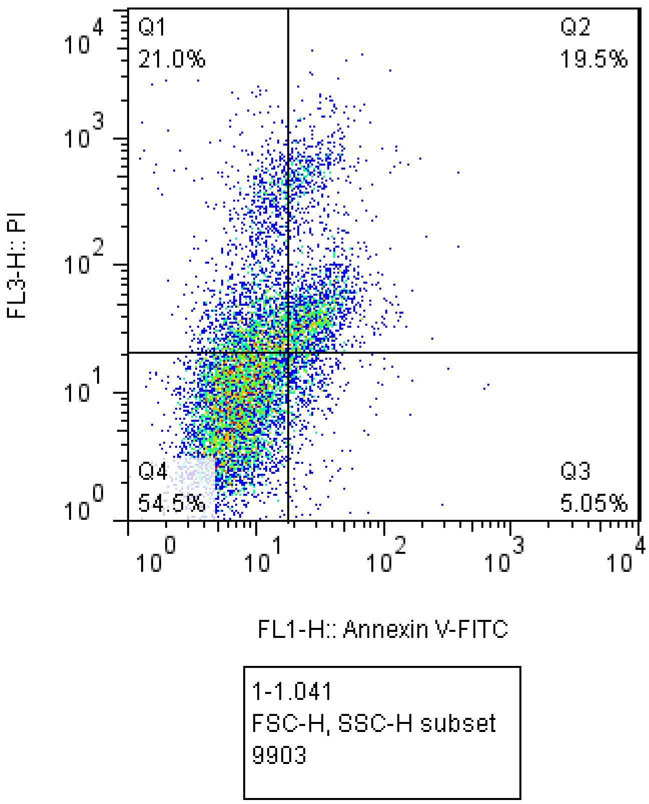

Dox 1

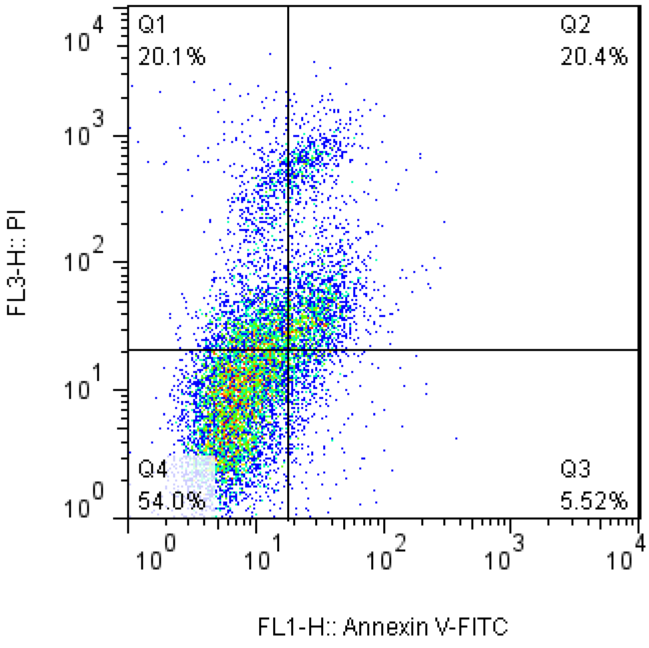

1-2.042
FSC-H, SSC-H subset
10525

Dox 2

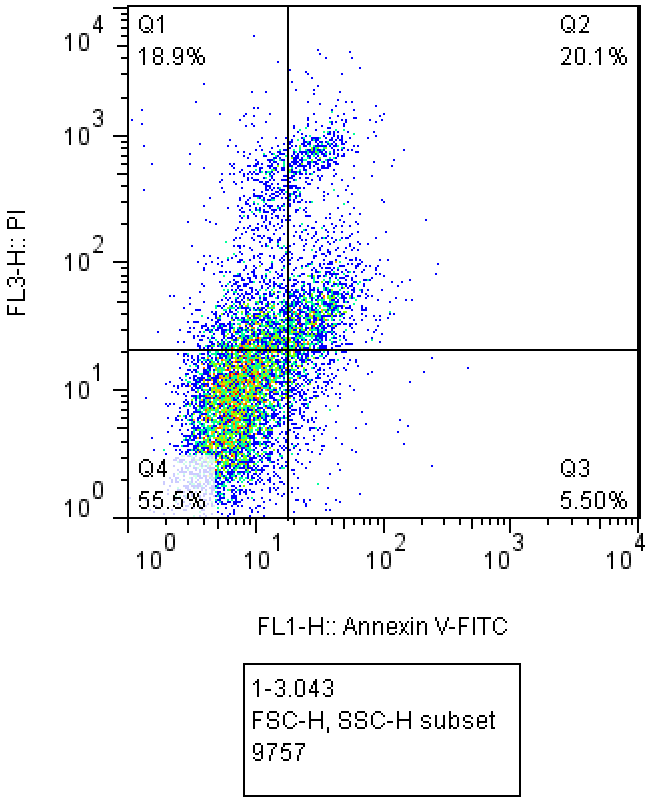
Q1
18.9%
Q2
20.1%
Q4
55.5%
Q3
5.50%
FL3-H:: PI
FL1-H:: Annexin V-FITC
1-3.043
FSC-H, SSC-H subset
9757

Dox 3

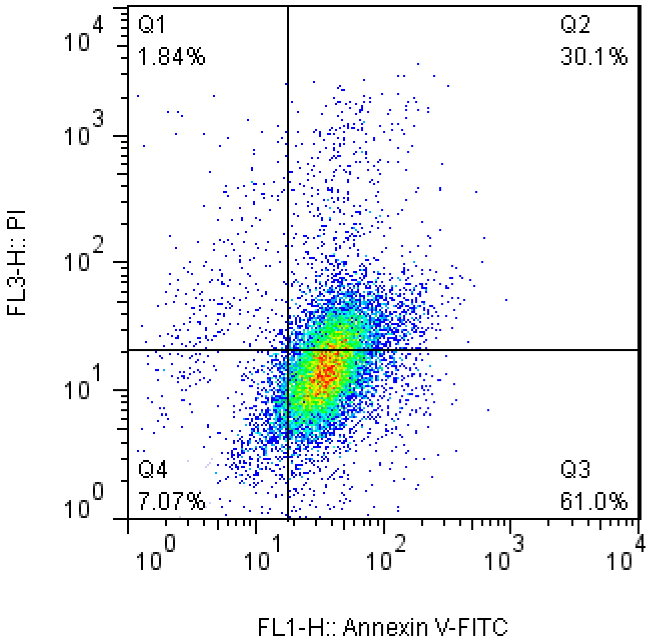

2-2.046
FSC-H, SSC-H subset
14816

NE 1

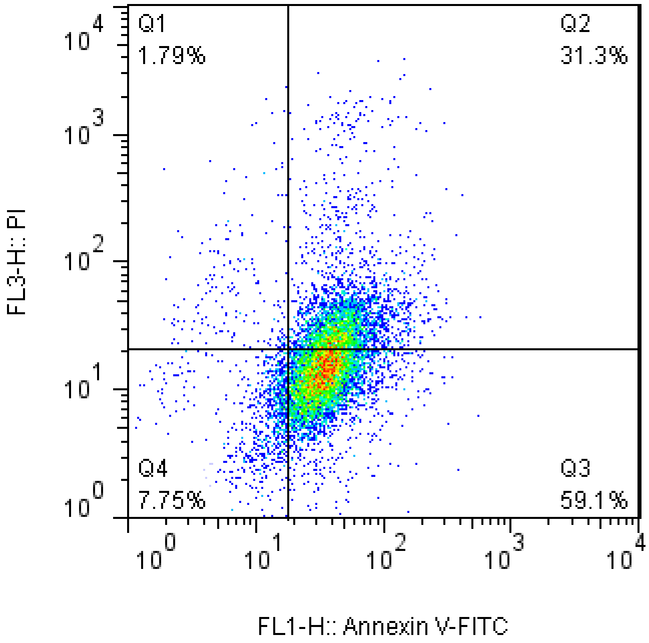

2-3.047
FSC-H, SSC-H subset
10759

NE 2

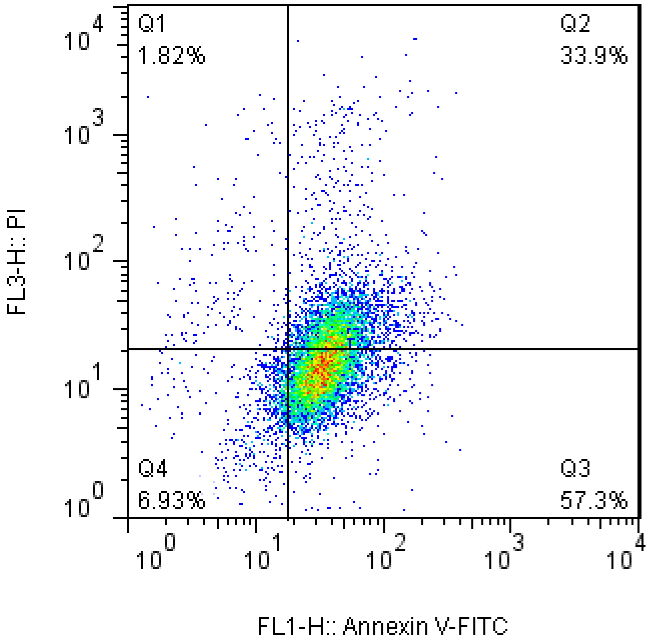

2-4.048
FSC-H, SSC-H subset
10249

NE 3

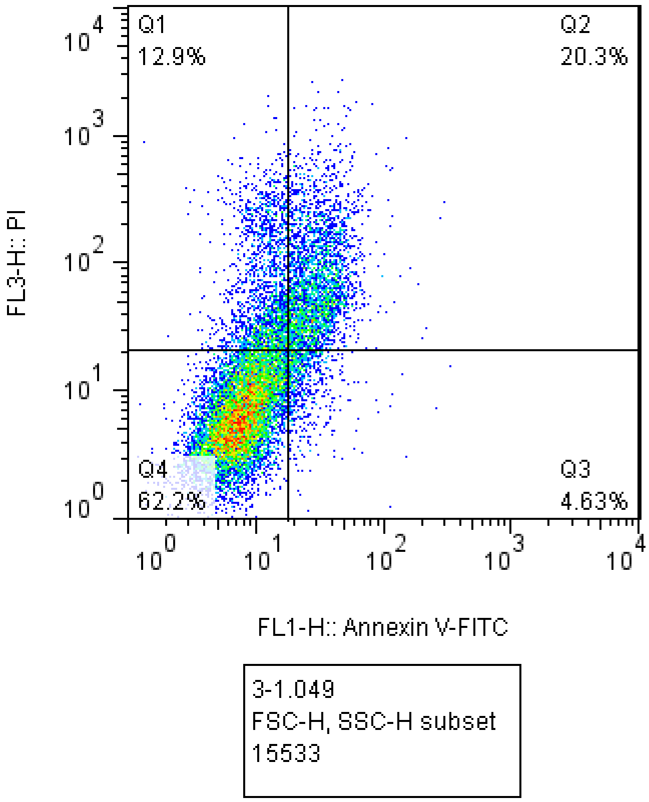
Q1
12.9%
Q2
20.3%
Q3
4.63%
Q4
62.2%
FL3-H:: PI
FL1-H:: Annexin V-FITC
3-1.049
FSC-H, SSC-H subset
15533

Extract 1

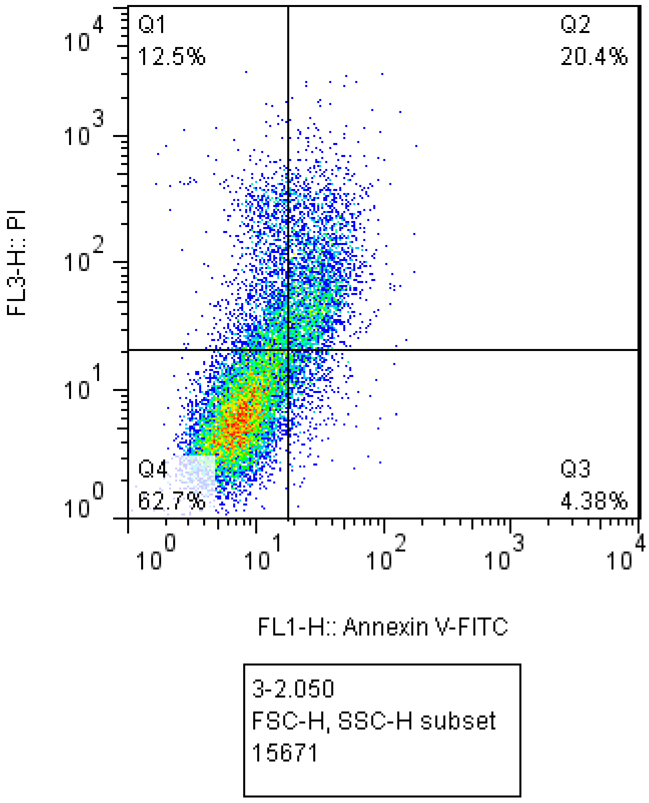
Q1
12.5%
Q2
20.4%
Q4
62.7%
Q3
4.38%
FL3-H:: PI
FL1-H:: Annexin V-FITC
3-2.050
FSC-H, SSC-H subset
15671

Extract 2

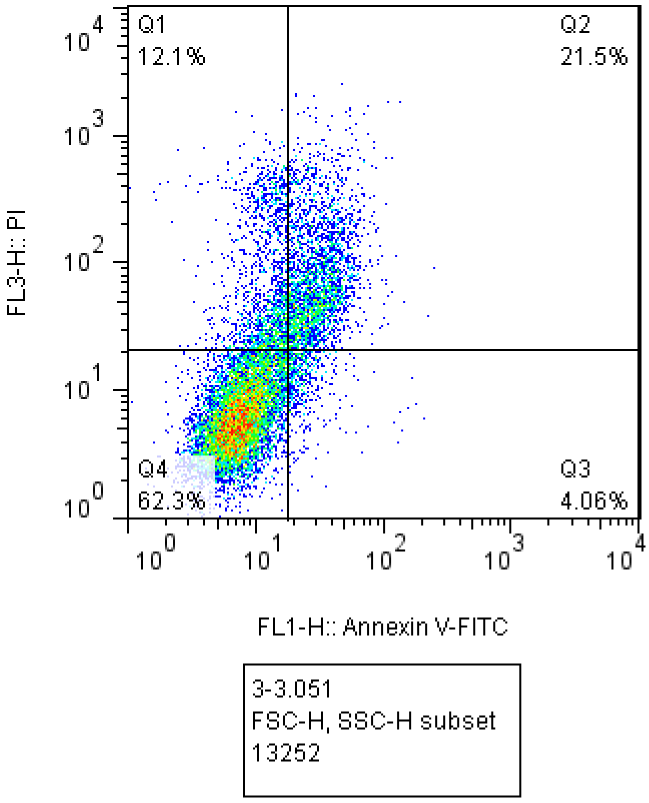
Q1
12.1%
Q2
21.5%
Q4
62.3%
Q3
4.06%
FL3-H:: PI
FL1-H:: Annexin V-FITC
3-3.051
FSC-H, SSC-H subset
13252

Extract 3

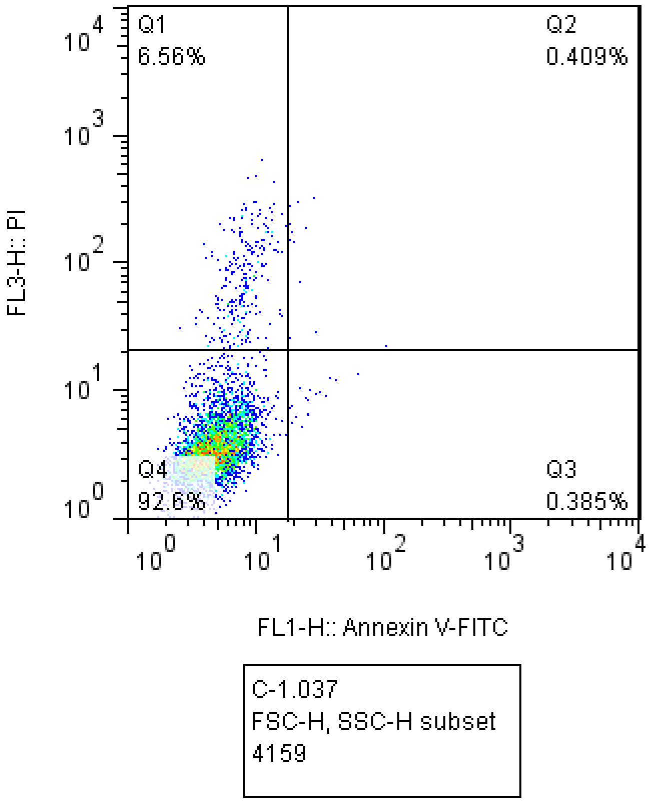
Q1
6.56%
Q2
0.409%
Q3
0.385%
Q4
92.6%
FL3-H:: PI
FL1-H:: Annexin V-FITC
C-1.037
FSC-H, SSC-H subset
4159

Control 1

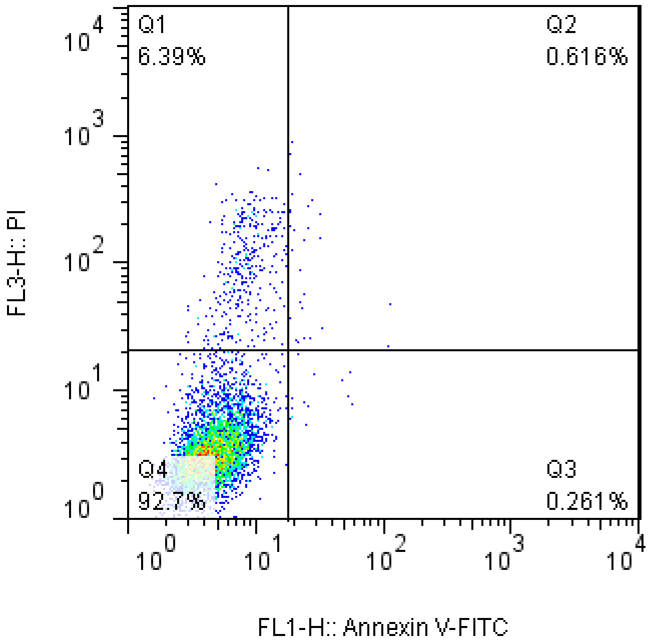

C-2.038
FSC-H, SSC-H subset
5356

Control 2

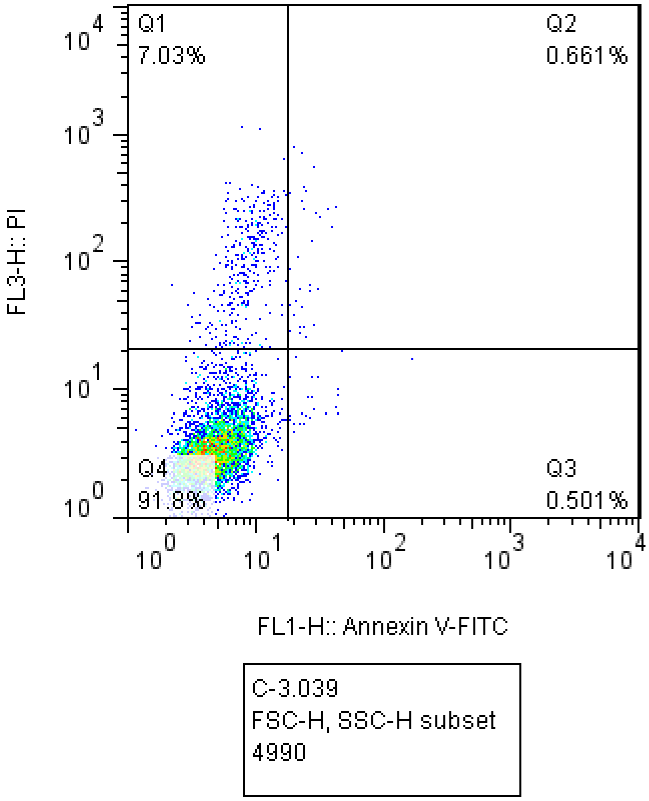
Q1
7.03%
Q2
0.661%
Q4
91.8%
Q3
0.501%
FL3-H:: PI
FL1-H:: Annexin V-FITC
C-3.039
FSC-H, SSC-H subset
4990

Control 3
