# Supplementary material for: Enhanced anticancer activity of nanoemulsified cardamom extract via modulation of apoptosis- and lncRNA-associated pathways in colorectal cancer cells
Source: Biochem Biophys Rep. 2026 Feb 1;45:102455. doi: 10.1016/j.bbrep.2026.102455 (PMC12882662; doi:10.1016/j.bbrep.2026.102455)
Supplement: Multimedia component 2 [file mmc2.pdf]

Gating strategy

The gating strategy was performed step by step. First, cells were selected based on SSC/FSC scatter to eliminate debris and non-cellular particles. Then, single cells (singlets) were isolated. Dead cells were identified using PI staining and excluded from the analysis. Finally, the live cell population was evaluated using Annexin V and PI: PI^+/Annexin V^- cells were considered early apoptotic, PI^+/Annexin V^+ cells were classified as late apoptotic or necrotic, and PI^-/Annexin V^- cells were considered viable. Gating boundaries were determined using negative controls and single-stained samples.

- SSC/FSC: To remove debris and select the cell population
- FSC-A vs. FSC-H: To remove doublets and select singlets
- PI: To exclude dead cells
- Annexin V/PI: To determine apoptosis stages
- Negative and single-stain controls: To define gating boundaries

A step-by-step gating procedure was used to identify apoptotic cell populations:

1- Removal of debris and cellular remnants based on forward and side scatter (FSC/SSC).

2- Selection of single cells using FSC-A versus FSC-H plots to exclude doublets.

3- Exclusion of dead cells by PI staining.

4- Final analysis using Annexin V versus PI plots to distinguish viable cells (PI^-/Annexin V^-), early apoptotic cells (PI^+/Annexin V^-), late apoptotic or necrotic cells (PI^+/Annexin V^+), and dead cells (PI^-/Annexin V^+).

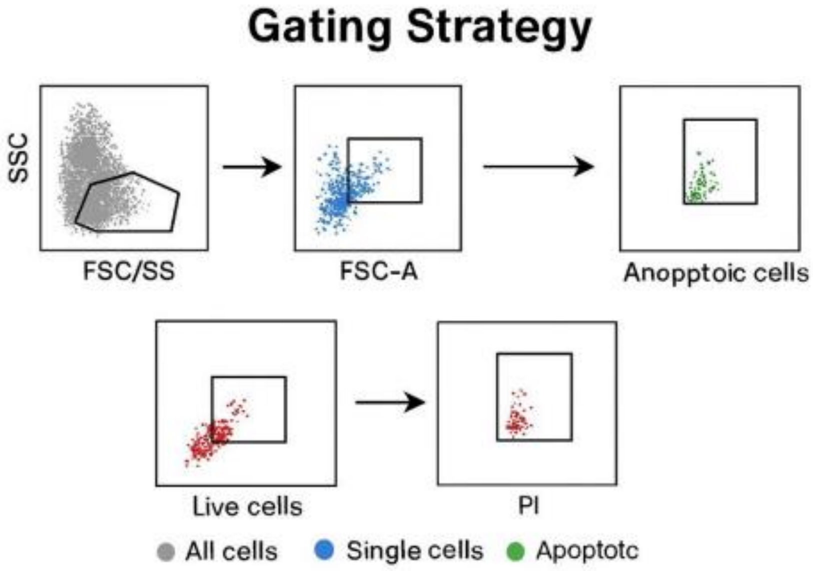

Compensation settings

To correct the spectral overlap between Annexin V-FITC and PI, compensation settings were adjusted using single-stained controls. The compensation matrix was automatically calculated and applied by the FlowJo software. The accuracy of compensation was confirmed by examining the position of single- and double-stained populations in dot plots.
